# Getting back on track after treatment of cancer: A qualitative interview study of cancer survivors’ experiences

**DOI:** 10.1371/journal.pone.0313984

**Published:** 2025-01-09

**Authors:** Doris van der Smissen, Marjolein Lugtenberg, Manon Enting, Laurens Beerepoot, Floortje Mols, Evelien Brouwers, Dareczka Wasowicz, Margot Joosen

**Affiliations:** 1 Tranzo, Scientific Center for Care and Wellbeing, Tilburg School of Social and Behavioral Sciences, Tilburg University, Tilburg, The Netherlands; 2 Department of Dermatology, Erasmus MC Cancer Institute, University Medical Center Rotterdam, Rotterdam, The Netherlands; 3 Department of Internal Medicine, Elisabeth-TweeSteden Hospital, Tilburg, The Netherlands; 4 Center of Research on Psychological Disorders in Somatic Diseases, Department of Medical and Clinical Psychology, Tilburg University, Tilburg, The Netherlands; 5 Department of Research, Netherlands Comprehensive Cancer Organization, Utrecht, The Netherlands; 6 Department of Surgery, Elisabeth-TweeSteden Hospital, Tilburg, The Netherlands; University of Pretoria, SOUTH AFRICA

## Abstract

**Objective:**

An increasing number of people resumes life after cancer treatment. Although the (long-term) side-effects of cancer and its treatment can be significant, less is known about the impact on cancer survivors’ participation in daily life. The aim of this study was to explore the common experiences of cancer survivors in resuming life after treatment.

**Methods:**

A semi-structured interview study was conducted among 22 cancer survivors having a favorable prognosis after treatment. Purposive sampling was used to select a variable sample in terms of diagnoses (e.g. breast cancer, colorectal cancer, melanoma), age (18–77 years), and time after diagnosis (0–7 years). Interviews were audio-taped, transcribed verbatim and analyzed in a thorough thematic analysis.

**Results:**

Four main themes were identified. First, cancer survivors reported an *emotional fallout episode* to often follow treatment, which was characterized by a loss of direction and control due to discontinuation of medical care, decreased support from healthcare professionals and their social network, and an emotional set back. Second, survivors experienced *challenges with getting back on track* such as the impact of impaired physical and cognitive functions, and challenges and uncertainty related to work and finances. Third, in *coping with changes and regaining trust* they tried to find a balance between dealing with patient stigma and fear of recurrence on the one hand, and maintaining a positive mindset on the other hand. Fourth, the disease often led to *re-evaluating values in life*, *health and work*, which included realizing and accepting change and having a more conscious outlook on life.

**Conclusion:**

In resuming life after treatment, cancer survivors experience several challenges and changes in values in different life domains that extend beyond the specific diagnosis. To improve supportive care, healthcare professionals should focus on the (changed) individual needs and values of survivors in the domains considered relevant to them.

## Introduction

Cancer rates are rapidly growing worldwide [1]. Due to advances in early detection of cancer, and new treatment options such as immunotherapy and targeted therapy, the number of cancer survivors also continues to grow [2]. In the Netherlands, 126,987 persons were diagnosed with cancer in 2022 and the 5-year and 10-year survival rate were respectively 66% and 57% [3]. As a result, an increasing number of people resumes life after cancer treatment [2].

Previous quantitative studies have demonstrated that cancer survivors may experience several problems, such as persistent or recurring physical complaints as well as psychological problems [3–5]. These studies primarily focus on health and long-term effects of treatment using a quality of life approach [3–5]. However, a cancer diagnosis may also change the way individuals view themselves and what they deem important in life. This may have an effect on various life domains, such as work, family, leisure and community [5–10]. This is especially relevant in the transition phase from patient to cancer survivor and participating citizen. This broadened perspective aligns with the International Classification of Functioning, Disability and Health (ICF) model [11], which stems from its non-diagnostic, functionally focused, interactive, and contextually dynamic characteristics [11, 12]. Physical, social and emotional effects of cancer depend on the context, and differ between cancer survivors.

Few qualitative studies have explored the experiences of cancer survivors in-depth using this broad perspective on functioning. These studies have focused on survivors of specific cancer types such as melanoma [8], and head and neck cancer [9], or specific patient groups such as those with advanced breast cancer, lung cancer or melanoma who received long-term treatment [8, 13, 14] or specifically on patients with chronic cancer-related fatigue [15]. These studies highlight several important themes among cancer survivors, of which some seem to be disease or treatment specific, such as dealing with a (favorable or unfavorable) prognosis switch [8, 13, 14]. Other themes such as dealing with uncertainty and fatigue, appear to be relevant in multiple studies, and may therefore extend beyond specific cancer diagnosis [8, 9, 13–15]. Although the beforementioned studies focus on experiences of cancer survivors in resuming life after treatment per type of cancer diagnosis, generic and overarching experiences of cancer survivors in resuming their life after treatment in terms of participation, are yet unknown.

Therefore, the aim of this study was to explore the common experiences of cancer survivors in resuming life after cancer treatment. In doing so, we focus not only on the disease and physical functioning, but on the person having the disease, with attention for personal beliefs, values, preferences and goals [16].

## Materials and methods

### Study design

A qualitative study design using semi-structured interviews was considered most suitable to gain an in-depth understanding of cancer survivors’ experiences with resuming their life after treatment and (changes in) values and goals [17]. The advantage of individual interviews as opposed to focus groups is that these are considered most appropriate for sensitive topics [18].

The Consolidated Criteria for Reporting Qualitative Research (COREQ) [19] were followed during this study to correctly conduct and report the study. The Ethics Review Board of the School of Social and Behavioral Sciences of Tilburg University approved this study [RP576], as well as the Board of Directors of the Elisabeth-TweeSteden hospital (ETZ).

### Selection of participants

We aimed to include cancer survivors treated with curative intent with a favorable prognosis, who were treated at the Elisabeth-TweeSteden hospital (ETZ) in Tilburg in the South of the Netherlands. This non-university teaching hospital belongs to an association of top-tier hospitals in the Netherlands, providing high-level specialized care while contributing to (university level) medical training of healthcare professionals. We used purposive sampling [18] and aimed to include a variable sample of cancer survivors regarding the following characteristics: diagnosis (including breast cancer, colorectal cancer, and melanoma); sex; age (18 to 77 years); and time after diagnosis (0 to 7 years). Eligible participants were recruited by their attending physician; they received a patient information letter consisting of information about the study. Potential participants could sign up through an online questionnaire in Microsoft Forms in which they were asked for contact information, background characteristics, and for consent to participate. A researcher contacted the participants by phone or email to schedule the interview. The interviewees were offered a gift card of €25 in exchange for their participation. After 22 interviews, thematic saturation (see Data analysis) [18, 20] was reached and recruitment of patients ended. Participants were recruited from September 2021 to March 2022.

### Data collection

The semi-structured interviews were conducted face-to-face at a location of the participant’s preference, i.e. at Tilburg University, at the participant’s home, or online via Microsoft Teams. Of the 22 interviews, 20 were conducted face-to-face, and two were conducted online via Microsoft Teams. In case interviews were conducted online, participants were asked in advance through an online form in Microsoft Forms to read the informed consent letter. In the online form, they were asked whether they understood the text and agreed to participate in the study. In case they agreed, they were asked to provide online informed consent by checking a checkbox in the online form.

Prior to the interview, the interviewer provided information on the study. In case of a face-to-face interview, the participant signed the informed consent form. Subsequently, the interviewer conducted the interview using a predefined interview guide, see S1 Appendix. The interview guide addressed cancer survivors’ experiences with resuming their life after treatment (including experienced problems, experienced positive aspects, impact of the disease in several life domains and on relatives), and (changes in) values and goals. The interview guide was pilot tested with a patient expert by experience, who was treated for cancer more than 5 years ago and who did not participate in this study. Following her feedback small adjustments were made to the topic guide. The participants were not familiar with the interviewer or with the study prior to the invitation for the study.

### Data analysis

The interviews were audio recorded and transcribed verbatim without identifiers. The transcripts were analyzed using a thorough inductive thematic content analysis method drawing on elements from grounded theory [21–23], including the cyclical process of data collection and analysis, using multiple phases of coding, constant comparison, and sampling until saturation [21–25].

Directly after the interviews, the interviews were summarized by the interviewer (DvdS). During the analysis process, the software program ATLAS.ti 22 was used. First, two researchers (DvdS and ME) read, re-read and openly coded the data of the first four transcripts, thereby inductively assigning initial labels to the data [21, 23]. They also checked and complemented the open codes provided by the other researcher. After the open coding of the four interviews, the two researchers (DvdS and ME) developed an initial coding tree from the unstructured list of open codes consisting of initial concepts and sub-concepts, which was discussed in the multidisciplinary team (DvdS, ME, ML, MJ). During the axial coding phase, the remaining interviews were simultaneously openly and axially coded [21, 23] using this coding tree, by assigning axial codes from the coding tree, and by identifying new connections and axial codes [26]. The coding tree was constantly adapted during this axial coding phase and discussed within the multidisciplinary research team. In the last phase, the selective coding phase, the multidisciplinary research team identified the linkages between the (sub) concepts, and identified main and subthemes. After 22 interviews, thematic saturation had been reached as we concluded that no new themes or subthemes in terms of common and overarching experiences of cancer survivors as a group (rather than per diagnosis) could be identified from additional data [18, 20]. Throughout the analysis process, we used the constant comparative method, comparing the emerging findings with new data [18, 27]. Two patient experts by experience, one who was treated for cancer more than 5 years ago and one who was still in treatment, provided feedback during a presentation of the findings. They were asked whether they recognized the themes or not, and were asked to reflect on these themes. They agreed with the themes that emerged from the interviews.

## Results

Of the 22 participants, 16 were female, and the mean age was 52.7 years (range 41–77 years) (see Table 1). Most participants (n = 13) were currently working, and others were partly on sick leave (n = 3), fully on sick leave (n = 3), unemployed (n = 2) or retired (n = 1). The included participants had various diagnoses in terms of cancer type and had been treated with different treatments. Most participants completed their treatment (n = 16) and 6 participants were in long-term treatment such as immunotherapy or hormone therapy.

**Table 1:** Participants’ characteristics.

ID	Sex, Age	Currently working	Diagnosis	Years after diagnosis	Finished or current treatments	Time since finishing primary treatment
P1	F, 43	Partly on sick leave	Breast cancer with metastases	0 to 2	Finished: surgery, chemotherapy, radiation Current: hormone therapy	10 months
P2	F, 52	Fully at work	Breast cancer	3 to 4	Finished: surgery, radiation	4 years
P3	F, 59	Fully at work	Breast cancer	0 to 2	Finished: surgery, radiation	11 months
P4	F, 67	Fully at work	Breast cancer	0 to 2	Finished: surgery, radiation	2 years
P5	M, 53	Fully on sick leave	Laryngeal cancer with metastases	0 to 2	Finished: surgery, chemotherapy, radiation, chemoradiation	4 months
P6	F, 59	Fully at work	Breast cancer	3 to 4	Finished: surgery, radiation	3 years
P7	M, 64	Fully at work	Colorectal cancer	3 to 4	Finished: surgery	2 years
P8	F, 61	Unemployed	Colorectal cancer	0 to 2 and 3 to 4	Finished: 2 surgical interventions	4 years
P9	M, 46	Partly on sick leave	Colorectal cancer	0 to 2	Finished: surgery, chemotherapy	3 months
P10	F, 58	Fully at work	Colorectal cancer	3 to 4	Finished: surgery	3 years
P11	M, 53	Unemployed	Stomach cancer	0 to 2	Finished: surgery, chemotherapy	4 months
P12	F, 52	Fully at work	Stomach cancer	0 to 2	Finished: surgery	1 year and 10 months
P13	F, 42	Partly on sick leave	Colorectal cancer	0 to 2	Finished: surgery	8 months
P14	M, 44	Fully at work	Colorectal cancer	3 to 4	Finished: surgery, chemotherapy	1 year and 9 months
P15	F, 46	Fully at work	Breast cancer and thyroid cancer	0 to 2	Finished: surgery, chemotherapy, radiation Current: hormone therapy	8 months
P16	F, 77	Retired	Melanoma	0 to 2 and 3 to 4	Finished: surgery	7 years
P17	F, 48	Fully on sick leave	Thyroid cancer	0 to 2	Finished: surgery Current: iodine treatment	9 months
P18	F, 49	Fully at work	Melanoma	0 to 2	Finished: surgery	10 months
P19	F, 23	Fully on sick leave	Thyroid cancer	0 to 2	Finished: surgery Current: iodine treatment	7 months
P20	M, 53	Fully at work	Melanoma	7	Finished: 4 surgical interventions	7 years
P21	F, 69	Fully at work	Melanoma	0 to 2	Finished: surgery Current: immunotherapy	8 months (currently on treatment)
P22	F, 41	Fully at work	Melanoma	0 to 2	Finished: radiation Current: immunotherapy	3 months (currently on treatment)

### Experiences of cancer survivors in resuming their life after treatment

We identified four main themes and 13 underlying subthemes of cancer survivors’ experiences in resuming life after treatment, which are described in detail below. The main themes and subthemes are presented in Table 2.

**Table 2:** Overview of the main themes and subthemes.

**1. Emotional fallout episode after treatment** • Loss of direction and control due to discontinuation of medical care • Decreased attention and support from social network • Emotional set back
**2. Challenges with getting back on track** • Impact of impaired functions on resuming daily life • Challenges related to work and uncertainty regarding finances • Dealing with impact of disease on close relatives
**3. Coping with changes and regaining trust** • Dealing with self/other imposed patient stigma • Fear of recurrence of disease, fear of death • Need to regain trust in body • Maintaining a positive mindset
**4. Re-evaluating values and goals in life, health and work** • Realizing and accepting change • More conscious outlook on life • Redefining values and goals in health, relationships and work

### 1. Emotional fallout episode after treatment

The first main theme is an ‘*Emotional fallout episode after treatment’*. This episode was mostly temporary in nature, and its start varied from directly after the end of the primary treatment to when survivors resumed their daily life. It included the following three subthemes: *Loss of direction and control due to discontinuation of medical care*; *Decreased attention and support from social network;* and *Emotional set back*.

• *Loss of direction and control due to discontinuation of medical care*: During their treatment, survivors indicated to have little time to reflect on their situation because of time-consuming treatment schedules. They felt they ‘just’ had to undergo the treatments and felt being lived. This so called ‘survival mode’ felt intensive but also provided a feeling of control, because patients lived from goal to goal during their disease trajectory. According to survivors, this intensive treatment schedule and associated support of healthcare professionals often disappeared quickly after treatment had finished, also referred to as ‘discontinuation of medical care’. Survivors indicated to experience loss of direction and control due to this discontinuation. They often missed guidance on how to resume their life after treatment. Some felt being left to fate after treatment, since they had no idea what to expect from the future and how to move forward.

Subtheme: Loss of direction and control due to discontinuation of medical care

*"Then it’s kind of an emotional set back*. *It’s weird because you go to the hospital every three weeks*, *you come out of that chemo miserable*, *someone says*: *it’s going well*, *hang on*. *And then that’s done*, *then you no longer*.* *.* *.*[have the support]*.*"–Man*, *46 years (patient 9)*

Subtheme: Loss of direction and control due to discontinuation of medical care

*“The current supportive care is very medically oriented*, *(*.* *.* *.*) in the form of mammograms*, *feeling [the breast]*, *hearing how it’s going*, *but those are very brief consultations of course*, *that gives something to hold on to and that’s fine if that’s okay*, *so to speak*. *But real supportive care as in psychological aftercare*, *(…) I think there could be more attention for that*, *because when the check-ups are done you are just left to fate in that respect*.*”–Woman*, *52 years (patient 2)*

• *Decreased attention and support from social network*: Similar to the experienced reduction of care from healthcare professionals, survivors often reported faded support and lack of understanding of close relatives after treatment. During the intensive treatment trajectory, survivors received much attention and support from their social network. After treatment, close relatives carried on with their lives and–according to survivors–they often expected the same from the survivor and had less attention for the survivor. This may be due to unawareness of survivors’ still ongoing problems and invisibility of their lasting symptoms. Although the perception that relatives carried on with their lives was generally perceived as difficult by survivors who were not always ready to resume their daily life (see main theme 2), it was also sometimes experienced as pleasant as it encouraged survivors to carry on with their lives as well.

Subtheme: Decreased attention and support from social network

*“The people around me*, *my friends*, *said*: *“If you need help*, *we will be there for you*!*” But actually*, *if I would need something*, *no one comes to help me*, *except [name friend]*, *who helps me*.*”–Woman*, *48 years (patient 17)*

Moreover, support from survivors’ social network was sometimes reported to be disrupted by COVID-19 measures, such as social distancing, which–according to survivors–resulted in intensified feelings of loneliness at home, or in the hospital, because family members were not allowed to accompany them.

Subtheme: Decreased attention and support from social network

*“The only thing I found difficult*, *is that during the COVID-19 pandemic*, *my husband was not allowed to come with me to the hospital*, *he had to drop me off in the morning and then I needed to sit there all alone in a chair*, *(…) that was because of the COVID-19 pandemic*.*”–Woman*, *49 years (patient 18)*

• *Emotional set back*: Because of the loss of control and the perceived discontinuation of support, survivors often reported experiencing an emotional set back after treatment, which sometimes occurred unexpectedly and often persisted for a longer period of time. During this emotional set back, survivors felt they were confronted with the emotionally demanding experiences of the diagnosis and treatment phases. They had yet to process these experiences, sometimes resulting in feelings of shock, disbelief, fear, guilt, sadness, distress, uncertainty and for some in psychological trauma. According to survivors, the experienced challenges with getting back on track after treatment (see main theme 2), influenced this emotional set back.

Subtheme: Emotional set back

*“It’s tough*, *you have to mentally process everything you’ve been through physically*. *(*.* *.* *.*) The moment your treatments start*, *you put your emotions to the background*. *After the treatment*, *the mental*, *emotional set back comes*. *(…) during those treatments you’re really in survival mode*, *but when that’s done you have that peace of mind [the realisation]*, *what actually happened*.*”–Woman*, *42 years (patient 13)*

### 2. Challenges with getting back on track

The second main theme ‘Challenges with getting back on track’, consisted of the following subthemes: *Impact of impaired functions on resuming daily life*; *Challenges related to work and uncertainty regarding finances*; and *Dealing with impact of disease on close relatives*.

• *Impact of impaired functions on resuming daily life*: According to survivors, dealing with impaired physical, mental, and cognitive functions posed difficulties in getting back on track after treatment and resuming daily life. This applied for example to taking care of their family, household chores, daily exercising (walking, cycling, recreative sports), work and social activities. They generally expected to be able and ready to resume their life as soon as they finished treatment. However, when they were not capable to do so, they often felt disappointed, mentally distressed, or they experienced feelings of failure or angriness, particularly as this made them feel that the disease still controlled their life after treatment. As a result, survivors often crossed their boundaries in this phase after treatment, by taking too little time for their recovery and resuming their activities too fast resulting in an increase of symptoms. Furthermore, survivors indicated finding it difficult to be dependent of others. They sometimes experienced feelings of guilt about becoming ill and wondered if they could have prevented this, e.g. by adopting a healthier lifestyle. According to survivors, having a less favorable and uncertain prognosis, an intensive treatment trajectory, illness because of the disease, or experiencing side effects of treatment contributed to a greater amount of psychological distress and subsequent impact. A quick diagnosis and treatment on the other hand, appeared to contribute to a faster recovery and generally lower impact on daily live, according to survivors. These survivors generally indicated to have less difficulty with resuming their daily activities again.

Subtheme: Impact of impaired functions on resuming daily life

*"At a certain point the treatments are finished (…)*, *and you think*: *okay*, *we’re ready to go again*. *But then you’re actually not ready*, *because your body isn’t ready yet*. *(…) That is really frustrating*.*”–Woman*, *41 years (patient 22)*

• *Challenges related to work and uncertainty regarding finances*:

Some survivors indicated that they were able to gradually get back to work after treatment. However, due to physical, mental, and/or cognitive impairments, survivors often needed to stop working temporarily or were unable to return to work, either in their previous or a new job. This generated much uncertainty according to survivors, and they often missed to work towards a goal because of this. Survivors often experienced long-term symptoms that complicated working, such as loss of energy or lack of concentration. Whereas survivors were generally motivated to work before the onset of their disease, they sometimes felt demotivated to return to work after treatment. This demotivation was fed by insecurities due to limited functional ability and limited support and understanding of their employer or colleagues. Furthermore, they experienced worries about the prolongation of their job contract. When they did lose their job during or after their sick leave, they indicated to feel disappointed and let down by their employer.

Subtheme: Challenges related to work and uncertainty regarding finances

*"I thought*: *well now it’s finished [the treatment] and then I’ll go back to my work*. *But I can’t do it and I’m fighting so hard*, *to be able to do it all again but (*.* *.* *.*) I can’t have my life as before*. *That also worries me*, *especially the financial part*. *I am in my second year of sick leave and in February the employer can actually fire me*. *In the end it’s very businesslike*. *Yes*, *that worries me a lot*.*"–Woman*, *43 years (patient 1)*

Some survivors experienced a lack of support from their employer. Recovery and return to work was experienced as difficult when they felt pushed and pressured to return to work by their employer and occupational physician. They indicated that the expectations of the employer and occupational physician were often too high. However, when the employer was supportive and showed understanding, the survivor indicated this to be helpful in their recovery. For instance, by providing the flexibility to recover from their disease in their own pace, and showing empathy for their situation. Survivors indicated to value little gestures from their employer during their sick leave such as a phone call to inform how they are doing, or drinking a cup of coffee together.

The (temporary) loss of a job and/or the dependence on sickness benefit sometimes resulted in financial uncertainty or problems, for instance by increased healthcare costs and the inability to get a mortgage after the disease.

Subtheme: Challenges related to work and uncertainty regarding finances

*“So then you get confronted with the strained housing market*, *but well it’s not even feasible [to buy a house] because if you have sickness benefits you can’t even get a mortgage*, *because your income is not taken into account*. *That [these concerns] can be added as well*.* *.* *.* *.*”–Woman*, *42 years (patient 13)*

• *Dealing with impact of disease on close relatives*: Besides the difficulty in dealing with the impact of the disease on their own lives, survivors often reported that it was also difficult to deal with the large impact of the disease on their close relatives. Relatives often felt fearful, sad, powerless and were afraid that the patient/survivor would die. Some survivors felt that the impact of the disease was even larger for their close relatives than for themselves. Therefore, survivors sometimes felt worried about their close relatives, and put themselves aside, to focus on their relatives instead. For example, the disease was often perceived to have a large impact on their children, particularly when children found it difficult to talk about their emotions.

Subtheme: Dealing with impact of disease on close relatives

“*My daughters didn’t talk about it [the disease] because then they noticed that especially my wife got very sad*. *And once in a while I would ask how she was doing and then they would break down and would start crying and then you hug them*, *but actually it never came to a normal conversation because the moment it happens they get emotional*. *And of course*, *they don’t start talking about it*, *because they know that mom gets very emotional then*. *(…) Yes*, *that has always been a struggle*.*”*–Man, 53 years (patient 5)

Moreover, similar to the survivor, the partner tended to go into survival mode during treatment as well, according to survivors. Some partners decided to temporarily stop working during treatment, because they needed to take care of the survivor. Similar to survivors, partners sometimes experienced an emotional fallout episode after treatment. Reasons for such an emotional fallout episode were unprocessed feelings of the disease (trajectory) and their long-term role as a caregiver, during as well as after the disease. In addition, survivors mentioned that partners often experienced psychological symptoms, sometimes even leading to burn-out. Survivors indicated that the partner was often forgotten by their social network and kept their emotions to themselves to not burden and protect their ill partner. At the same time, similar to survivors, relatives often experienced a changed view on life; they gained awareness of the vulnerability, preciousness and shortness of life (see theme 4: Re-evaluating values and goals in life, health and work).

Subtheme: Dealing with impact of disease on close relatives

*“My wife had a severe burnout after I got ill*. *(…) she had to take care of me and I couldn’t do anything in those six months*, *and she experienced a lot of stress*. *Then I recovered (…) And then she fell into that hole [emotional fallout episode]*. *She really had a really tough time*.*”–Man*, *44 years (patient 14)*

### 3. Coping with changes and regaining trust

Coping with changes and regaining trust was the third main theme, consisting of the following subthemes: *Dealing with self/other imposed patient stigma; Fear of recurrence of disease*, *fear of death; Need to regain trust in body;* and *Maintaining a positive mindset*:

• *Dealing with self/other imposed patient stigma*: Survivors indicated to have to deal with both self-imposed patient stigma as well as social patient stigma imposed by others, which were linked to one another. They often had negative attitudes about their own condition (self-stigma). Survivors did not want to be perceived as a cancer patient and weak. They sometimes downgraded their problems, because they felt they had no right to complain about their situation, and therefore kept a positive attitude with regard to their disease. They also talked less with their relatives about the disease because they did not want to burden them with their story. Whereas survivors overall did not want to be seen as a patient and ask for help to their social network, they also acknowledged that downgrading their problems resulted in their social network assuming that resuming their life was going well. Others perceived themselves as being a patient for the rest of their lives, and felt that this was linked to their annual check-ups in the hospital and to long-term treatments (hormone therapy).

Subtheme: Dealing with self/other imposed patient stigma

*“I don’t want people to see me*, *or see myself as that person with cancer*. *I want to remain just [name]*, *who used to be ill*. *(*.* *.* *.* *..*) but (…) now that I am at work and running into things again I think*: *oh yes*, *I am still ill*, *(…) the one with cancer*. *(…) Yes*, *you still notice this in daily life*.*”–Woman*, *23 years (patient 19)*

Survivors also experienced stigma imposed by their social network, i.e. perceived negative attitudes from their social network about their condition. Still being seen and treated as a patient by their social network (e.g. by continuously talking about the disease), and therefore to be frequently confronted with their disease, was perceived as difficult when trying to resume their life. At the same time, survivors were sometimes expected by their social network to not talk about their (former) disease, for instance by their employer to not talk about their situation to clients, which made them feel misunderstood.

Subtheme: Dealing with self/other imposed patient stigma

*“Yes I have the experience that I can talk [about the disease]*, *but I sometimes hear fellow cancer survivors around me saying*: *“well*, *they never ask me how I am doing”*. *And then I think*, *yes*, *these are very small words*, *but they are so important*. *They are very important*.*”–Woman*, *69 years (patient 21)*

• *Fear of recurrence of disease*, *fear of death*: Despite their overall favorable prognosis, survivors often experienced a fear of cancer recurrence and sometimes fear of death. Particularly before check-ups at the hospital, survivors generally felt nervous and anxious because of this fear for recurrence. For some survivors, this fear of recurrence was very prominent and therefore negatively influenced resuming their life. Other survivors could cope with the fear quite well and they could accept the uncertainty of possible recurrence. Also, survivors were more conscious of physical symptoms as possible indicators for the disease. As a result, they constantly checked their body for symptoms of recurrence and focused more on prevention of the disease (e.g. reducing sun bathing and healthy eating and exercising).

Subtheme: Fear of recurrence of disease, fear of death

*"…but also the uncertainty of*: *if I feel anything [physical symptoms]*, *I am rather afraid that it could be something wrong again [recurrence]*. *Yes*, *the uncertainty of*, *how do we get out of this in the best way*, *also with the family*.*"–Woman*, *43 years (patient 1)*

• *Need to regain trust in body*: Survivors often felt that their body had let them down, felt insecure about impaired body functions, and needed to regain trust in their body. Often survivors’ bodies had changed, for instance by scars, losing a breast, losing organs (such as the thyroid or esophagus) or getting a stoma. This sometimes led to a lower self-image regarding their appearance and a lower trust in their body. Other survivors could better accept their changed body, for instance due to their positive attitude. Generally, survivors experienced an increased consciousness that one can become ill suddenly, and therefore felt vulnerable. Therefore, survivors often needed time to process and realize that they survived the disease as well as to regain trust in their body again.

Subtheme: Need to regain trust in body

*“You have to start all over again*. *You regain a little bit of confidence in your own body*, *and you have to learn to enjoy little things again*.*”–Woman*, *42 years (patient 13)*

• *Maintaining a positive mindset*: Besides feelings of fear and uncertainty, survivors often stressed the importance of maintaining a positive mindset. According to survivors, a positive, faithful, optimistic and level-headed attitude helped them to accept their former disease and its consequences. It also helped them to cope with the uncertainty as a consequence of the disease. Helping attitudes were focusing on surviving the disease, accepting their situation and letting go of things, instead of focusing on things they are not capable of doing anymore. Some survivors received treatment from a psychologist to cope with the disease. Feelings of hope and optimism often alternated with feelings of fear of recurrence, and their intensity varied over time.

Subtheme: Maintaining a positive mindset

*“I’m a positive person so that has helped a lot in my recovery*, *yes*.*”–Woman*, *58 years (patient 10)*

In addition, putting things into perspective also helped to cope with the impact of the disease and the experienced uncertainty, according to survivors. The thought of having an equal probability of having cancer as any other person, helped to put their fear of disease recurrence into perspective. Furthermore, they felt supported by realizing that they are not the only one with the disease and by realizing that any person can get the disease. Comparing their own situation to the situation of others with a more severe disease trajectory, realizing that their own diagnosis could have been worse, helped survivors to put their fear into perspective. Also worrying less about small daily problems and irritations, obligations and relationships was stressed by survivors as a way of coping with the changes. This ability to put things into perspective was linked to their increased awareness of the preciousness and vulnerability of life (see main theme 4).

Subtheme: Maintaining a positive mindset

*" I went in there [the hospital]*, *(…) and you see other people… (…) What I have is bad*, *but there are worse things… And if those people can handle it*, *then you can too*.*” Woman*, *41 years (patient 22)*

### 4. Re-evaluating values and goals in life, health and work

Re-evaluating values and goals in life, health and work was the fourth main theme, consisting of the following subthemes: *Realizing and accepting change; More conscious outlook on life* and *Redefining values and goals in health*, *relationships and work*.

*•* *Realizing and accepting change*: Survivors indicated to realize at some point after treatment that their life needed to be rearranged and would not be the same as before their disease. They explained that they needed to prioritize more and set boundaries. Although this was often perceived as a difficult process, it was also experienced as helpful and pleasant as they were often unable to prioritize themselves before the disease.

Subtheme: Realizing and accepting change

*“Well I can prioritize better*, *also for example at work*, *I can quickly be like*: *well I’m really not going to spend any more time and energy on that*, *because I already know how it works*, *because of my experience*, *but also because you just know*, *this doesn’t make any sense anyway to start wasting your energy on this when things are going on or so at work*.*”–Woman*, *42 years (patient 13)*

*•* *More conscious outlook on life*: Generally, survivors indicated that their outlook on life had changed after the disease and treatment. They explained that they were now ‘living in the moment’ more often, and were more conscious and appreciative of the preciousness of life and their vulnerability after having this life-threatening disease and facing the finitude and shortness of life. Consequently, survivors indicated to enjoy life more consciously, and to do more of the things they love and get energy from, such as practicing their hobby. Furthermore, they indicated to prioritize themselves more, and put small problems into perspective resulting in less hurry and worries and more peace of mind. This was experienced as a process in which survivors needed to learn to enjoy their life after the disease, while processing their experiences with the disease.

Subtheme: More conscious outlook on life

*" I’ve started to set different priorities and appreciate other things that I used to take for granted*, *now I appreciate them more*. *(…) The whole thing [the disease] hasn’t brought me so much bad actually*, *maybe more good than bad*.*”–Man*, *53 years (patient 20)*

*•* *Redefining values and goals in health*, *relationships and work*: According to cancer survivors, their disease and the realization that their life is vulnerable and precious, often made them redefine their life values and goals. Survivors valued their health (even) more than before treatment. They realized that good health is not guaranteed and some even believed that they got the disease to learn to take better care of themselves, such as taking rest and healthier behaviors.

Subtheme: Redefining values and goals in health, relationships and work

*"I never really took good care of myself (…) like eating healthy*, *good sleeping patterns*, *taking rest when your body demands it*, *those things*. *I was always going on and on and on (…) I never really took rest*. *(…) I’m now trying to find that connection with my body again*, *because I know now how important that is*. *I think if I hadn’t gotten this disease then I would have just kept going*.*"–Woman*, *23 years (patient 19)*

Survivors also indicated to experience increased awareness of the importance of relationships. Not only did they get to know themselves and their relatives better, they also felt closer to and appreciated their family and social network (such as neighbors, sport contacts) even more than before treatment. Furthermore, more than before the disease, survivors realized that their family is most important in life and more important than work, resulting in survivors putting family first more often. Hence, they often felt that their relationships were enriched as a consequence of the disease. Furthermore, survivors mentioned to worry less about their relationships, and felt better able to let go of negative relationships. However, the disease could also negatively influence relationships, resulting in problems with and ending of (romantic) relationships.

Subtheme: Redefining values and goals in health, relationships and work

*“It has absolutely deepened my relationship with my partner*, *the relationship with my children… even though this has happened I have not been any more unhappy in that whole period than I would have been otherwise*, *of course I would have liked to skip it*, *but on the other hand it has also just brought me a lot*.*”–Woman*, *45 years (patient 15)*

Survivors also re-evaluated the importance of work in their life. After treatment, survivors often tended to worry less about their work and daily problems in general. They felt more motivated to better balance their work and personal life, and often considered work to be less important than before their disease. They sometimes decided to work less to be able to enjoy their personal life more. Others switched jobs because their illness made them realize they wanted a more enjoyable job. Furthermore, others mentioned to be able to do their work as a healthcare professional better than before the disease, because of increased understanding of what it feels like to be a patient.

Subtheme: Redefining values and goals in health, relationships and work

*“I think work-life balance*, *being able to take rest*, *exercise*, *eat healthy food is very important and also being able to enjoy myself*. *Enjoyment is really the most important thing*. *You just know it could have gone the other way*, *(*.* *.* *.*) it could have gone the wrong way*. *(*.* *.* *.*) Well*, *you never know*. *I could work myself to death for a few pennies to spend a little more*, *but I prefer to relax and enjoy life and go on vacation and do the things that I want to do*.*”–Man*, *44 years (patient 14)*

## Discussion

The present study aimed to explore the common and overarching experiences of cancer survivors in resuming life after cancer treatment. The results showed that cancer survivors experience several common challenges and associated changes in values in several life domains, including family, health and work. A (temporary) emotional fallout episode often follows treatment, after which survivors struggle with resuming their life in several life domains. The process of coping with all the experiences, changes associated with the disease and its treatment, and regaining trust, eventually leads to a re-evaluation of values in life, health and work.

An interesting finding of our study is the commonly experienced emotional fallout episode among cancer survivors. This emotional fallout episode is rarely studied in the scientific literature. Some previous studies do describe ‘emotional fallout’ among cancer survivors that have survived cancer for at least 5 years [28]. Here, it is described as survivors that keep experiencing psychological and psychosocial problems such as fear of recurrence, problems related to sexuality and body image, but it is also described that most survivors report overall emotional adjustment comparable to individuals without cancer [28]. In the current study, we identified an emotional fallout episode, that starts varying from directly after the end of the primary treatment to when survivors resumed their daily life. We also found that the partner of the cancer survivor may experience a comparable emotional fallout episode. This implies that awareness of this emotional fallout (episode) among cancer survivors, their relatives and healthcare professionals is important throughout the disease trajectory and after survival, to ensure appropriate and timely support both during and after finishing treatment.

The finding that cancer survivors experience several common challenges, that extend beyond the specific diagnosis, and associated changes in needs and values in several life domains aligns with the dynamic International Classification of Functioning, Disability and Health (ICF) model [11]. This model implies that the experienced impact of the disease depends on the context and may differ for each person [11, 12]. Rather than only focusing on the disease and symptoms, it is important to look at the individual experienced impact and associated (changed) needs and values in several life domains. This is also consistent with evolving definitions of “health” which focus not only on physical health, but also on the ability to self-manage and cope with challenges [29]. Several previous studies also found that cancer survivors described positive personal changes, for instance a different, positively changed perspective in life and improved personal growth [28, 30–32]. Our study adds to this, that survivors of different types of cancer may experience these changed values and a changed (more conscious) outlook on life within several domains such as health, relationships and work. Consequently, getting back on track often means getting on a different and more suitable track after treatment that fits survivors’ changed situation after treatment.

Furthermore, our results showed that in resuming life, cancer survivors often need to deal with patient stigma, both self-imposed as well as imposed by others. Cancer survivors struggled to find a balance between not wanting to be seen as a patient and therefore often downgraded their problems on the one hand, and on the other hand, wanting to receive recognition from themselves and others for their disease and its impact. This is consistent with a qualitative study which showed that stigma imposed by others may act as a barrier for cancer survivors to accept supportive care, as they tend to minimize their complaints [33]. Some quantitative studies showed that stigma imposed by others and self-stigma indeed exist among cancer survivors and stated that it should receive more attention [34–36]. The results of the current study highlight the need for increased attention to stigma by promoting recognition of the impact of the disease while at the same time focusing on individuals’ preferences and abilities in several life domains and society as a whole.

### Strengths and limitations

This study has several strengths. First, by including a purposively selected variable sample of cancer survivors in terms of types of cancer, types of treatments and time since diagnosis, we were able to provide an in-depth understanding of their common and overarching experiences that transcend specific cancer diagnoses in getting back on track after treatment. Moreover, by using a thorough thematic analysis approach drawing on elements from grounded theory [21, 23] (i.e. using multiple phases of coding, constant comparison, and sampling until saturation [21–25], the quality and robustness of our results were optimized. Another strength is the combination of face-to-face and online interviews dependent on the preference of the participant. Whereas face-to-face interviewing has the advantage of being able to observe social and non-verbal cues such as voice, intonation and body language, the disadvantages are the time to travel to the location of the interview for the participant and possibly feeling less comfortable than at home [37]. Online interviewing, on the other hand, has the advantage that participants can stay in their home setting, whereas social cues can generally be less easily observed [18, 37]. By adhering to the preference of the participant, the advantages of both methods are used, while their disadvantages are eliminated as much as possible.

This study also has several limitations. Participants were recruited via their medical doctor according to pre-defined selection criteria. However, physicians may have had a bias, for example by inviting patients who experience severe problems in resuming life or, on the contrary, experience few side effects, leading to a disproportional amount of extreme cases. However, by alternating the process of data collection and analysis, and thus by letting our initial findings guide further sampling leading to a diverse sample, we minimized this bias as much as possible. In addition, due to the regional nature of our project, participants were recruited in only one hospital, i.e. the Elisabeth-TweeSteden Hospital in Tilburg, the Netherlands. Although qualitative research is context-specific, we believe the identified overarching and common themes of cancer survivors in resuming life after treatment may well be transferable to other settings [38] within the Netherlands, and possibly even outside the Netherlands in case cancer care is organized in a comparable way. The themes identified in our study may also be transferable to other patient populations, as they may experience comparable challenges with getting back on track, for instance for survivors of physical trauma [39].

### Recommendations for future research

In this study we gained insight in the emotional fallout episode as experienced by cancer survivors and what it consists of. Future research could further examine this phenomenon in quantitative follow-up research. Specifically, more research into the prevalence of, and contributing factors (e.g. personality, coping style) for emotional fallout episodes among cancer survivors, as well as insight into how it can be prevented or adequately dealt with, is warranted. More in general, in order to provide appropriate supportive care that aligns with individual needs, it would be useful to explore common supportive care needs of cancer survivors by focusing not only on the disease and physical functioning, but on the person having the disease, with attention for personal beliefs, values, preferences and goals [16]. Because of their key role in the provision of appropriate care, exploring the perspective of healthcare professionals on adequate supportive care for cancer survivors in general, in addition to those of melanoma survivors [40], may be useful as well.

### Implications for practice

Our study implies focusing on the person beyond the patient and taking into account the (changed) individual needs and values in relevant life domains, may be beneficial in (supportive) cancer care. Improving awareness on the existence of the emotional fallout episode among cancer survivors, their relatives and healthcare professionals throughout the disease trajectory and the years after finishing treatment is important to ensure that cancer survivors and their relatives receive appropriate and timely support. Education of both patients and healthcare professionals about the importance of the different life domains may be a good means to facilitate addressing the needs of patients. Our results can serve as input for a (online) tool that facilitates the provision of supportive care in line with patients’ values, goals, and preferences.

## Conclusions

This study shows that cancer survivors experience several common and overarching challenges and associated changes in values in several life domains, including family, health and work, that extend beyond the specific diagnosis. To improve supportive care, healthcare professionals should focus on the person beyond the patient, taking into account (changed) values, goals and personal needs in the domains considered relevant to them. Especially since getting back on track after cancer treatment often means getting on a different and more suitable track, in which survivors adapt to their changed situation and needs.

## Supporting information

S1 Appendix Interview guide. (PDF)
