# Supplementary material for: Getting back on track after treatment of cancer: A qualitative interview study of cancer survivors’ experiences
Source: PLoS One. 2025 Jan 9;20(1):e0313984. doi: 10.1371/journal.pone.0313984 (PMC11717179; doi:10.1371/journal.pone.0313984)
Supplement: S1 Appendix — (PDF) [file pone.0313984.s001.pdf]

S1 Appendix. Interview guide.

Introduction

- Welcome
- Information about the study
- Written informed consent to participate and for audio recording the interview

Opening

- General experiences with having (had) cancer

Resuming life after cancer treatment

- Experienced challenges and/or encountered problems
- Experienced positive aspects or outcomes of the disease trajectory
- Experiences with being/feeling a (former) patient
- Experienced overall impact of the disease on life
    - E.g. symptoms and complaints, impact on functioning, norms and values

Impact of disease and/or treatment on loved ones

- Experienced impact of the disease on loved ones

Values and goals in life

- Important things in life (e.g. in work life, socially, mentally, physically)
    - Whether these things have changed since the diagnosis
- Specific values and goals in life
    - What they are and why
    - Whether they have changed compared to before the disease
